# Dr. Reynell Coates’ Contributions to Surgery: Note on the Means of Avoiding Excoriations of the Heel and Perineum in Fractures of the Lower Extremities, Treated by Permanent Extension

**Published:** 1842-05-28

**Authors:** Reynell Coates


MEDICAL EXAMINER.

NEW SERIES.

No. 22.] PHILADELPHIA, MAY 28, 1842. [Vol. I.

### Contributions to Surgery.

BY REYNELL COATES, M. D.

### *Note on the means of avoiding Excoriations of the Heel and Perineum in Fractures of the Lower Extremities, treated by Permanent Extension.*

Continued from p. 308.

Having now completed the review of the bandages for extension and counter-extension, noting those causes of excoriation which depend upon the construction of this portion of the apparatus, it is proper now to speak of the precautionary measures required in its management during the earlier weeks of the treatment.

One of the most important sources of difficulty will be found in the improper direction very often given to the bands. Instead of securing these in a direction corresponding with that of the axis of the injured limb, they are frequently carried towards the long lateral external splint or other proper appendage of the apparatus in a very oblique line,—thus placing them under circumstances of very great disadvantage for mechanical action.

When the method of Dr. Physick is adopted, the tapes of the gaiter or extending band may be occasionally seen to form an angle of forty-five degrees with the axis of the limb, owing to the use of chaff-bags of undue thickness between the splints and the limb, or a neglect of the proper adjustment of the block of Hutchinson, over which the tapes are passed before they are secured. This angular deviation sometimes results from the deficient height of the block, and sometimes from the notch being made altogether too deep. When acting at an angle of forty-five degrees, one-half the effective extending force of the band is lost, but the circular pressure which it exerts upon the heel and instep is not in any degree diminished by this loss; consequently, the tension of the tapes or band required to extend the limb to any given degree is then precisely doubled by the error of construction in the apparatus, and with it, *the danger of excoriation is doubled also.* Most absurd would it be to condemn the method of treating fractures of the lower extremities in the extended position on account of ulcerations produced by the ignorance or carelessness of the surgeon in regulating the height of the block. The proper direction may be readily given to the extending force in any case whatever, by deepening the notch with the saw, or filling it to a sufficient extent by inserting into it another block of suitable shape and size, and securing it there by glue or a brace of sprigs. The liability to the error just mentioned is less considerable when either of Dr. Hartshorne's apparatus is employed, as the foot board or the mortices of the cross piece are designed to occupy the middle spot between the two lateral splints; but we not unfrequently observe considerable loss of power even in this mode of treatment, from a neglect of the proper adjustment of the chaff-bags, which, if unduly thick on either side, will render the tapes or band more or less oblique. In fractures below the middle of the leg, when they require permanent extension at all, the neglect just mentioned inevitably produces a certain degree of angular deformity of the limb; but this result is not appreciable in fractures of the thigh.

Another considerable loss of power and a consequent increase of pressure on the perineum is sustained in consequence of the usual obliquity of the counter-extending band. It is not possible to devise any convenient apparatus upon the principles of Dessault or Physick which shall cause this band to act in a line exactly parallel with that of the axis of the limb. Some obliquity is unavoidable, but it should be reduced to a minimum. In the original apparatus of Dr. Physick, the external or long splint was provided with a stuffed crutch-like extremity adapted to the axilla. Whether this was designed by the illustrious inventor for the purpose of making counter-extension upon the axilla, or merely as a protection for the part, I am not prepared to state with absolute certainty; but, for many years prior to his death, the former was the received understanding of its intention, and for the latter, it is wholly unnecessary. It was discarded by its author very soon after its contrivance, although it still continues to figure in the descriptions of the apparatus in various surgical treatises. This crutch-like extremity increases the length of the splint by at least an inch—generally two inches—obliges the maker to form the mortices at more than that distance from the end next the axilla, thus increasing the obliquity of the band and, with it, the amount of pressure sustained by the perineum. It ought therefore to be universally rejected.

But owing to inattention to the laws of the resolution of forces, it has become customary to make the mortices at the distance of several inches from the upper end of the splint even after the rejection of the crutch-like extremity; thus, the obliquity of the band is unnecessarily rendered very considerable, and it is not surprising that excoriation of the perineum should be a frequent accident in the hands of surgeons so regardless of the first principles of mechanics as to tolerate this glaring error. The best mode of attaching the counter-extending band is to make the upper end of the splint very slightly concave, and to bore a single half inch auger hole within less than an inch of the centre of the curve, if the splint be made of firm wood:—if of ordinary pannel pine, an interval of full an inch may be required to give sufficient strength to the part. When applied, the posterior tape of the band should be passed through the hole, the anterior tape carried over the centre of the curved end of the splint, and the two tapes tied firmly in a double bow, on the outer side. If to these precautions we add that the length of the splint should always be sufficient to elevate its extremity as high as is convenient to the patient, making due allowance for the effect of the extending force in gradually straightening and stretching the almost inextensible band during the first few hours, we shall be possessed of all the requisite principles involved in the first dressing of a fracture with the least possible chance of excoriation from the action of the bands.

But it is not sufficient merely to economize the extending force by selecting bands of the right construction, and to apply them in such a manner as to act in the right direction. I have dwelt, in a former communication, (p. 164) upon the vast increase of the requisite effort and consequent pressure by any unnecessary delay in the first reduction of the fracture, and at the commencement of this paper, (p. 262) the influence of extensibility in the bands in producing the same result has also been mentioned. This difficulty is very often enhanced by over legislation on the part of the surgeon, or the officious intermeddling of nurses, inducing alternate relaxation and tightening of the bands, under the idea that such a measure is calculated to promote the comfort of the patient. Of all the causes of excoriation and ulceration this is the most frequent and powerful, and it is well deserving of more particular notice.

As I have had occasion to remark in one of my articles on myotomy and tenotomy, in the last volume of the Examiner, the human body is much more tolerant of uniform and permanent than it is of variable pressure, even when the latter is less considerable in degree. If a limb be regularly confined in turns of a moderately tight roller for half an hour, it will be found, on removing the bandage, that a universal engorgement of the superficial vessels takes place instantaneously, and the sensibility and irritability of the part are rapidly exalted. This is, obviously, owing to the fact that the mechanical support given to the vessels by the roller relieves their fibres from the necessity of vigorous contraction on their contents, and allows their tonicity to decline for want of exercise; so that, on the removal of the pressure, the vessels are incapable of completely resisting the *vis a tergo* of the circulation, and are therefore increased in calibre by hydrostatic pressure; more blood is containad in the part, but the vigour of the capillaries is not sufficiently diminished materially to retard the rapidity of the circulating current. Now, if pressure be renewed upon a part in this condition, it must act upon a surface strongly predisposed to irritation from slight causes, and, at each alternate increase and relaxation of the force, the danger must be enhanced.

The action of the extending band or gaiter almost always occasions con-considerable uneasiness to the patient within forty-eight hours of its first application. He complains of this—sometimes greviously—to his nurse or surgeon. This is a critical moment for those who would escape the vexations attendant upon an ulcerated heel or abrasion of the instep. Always supposing that the extension of the limb has been made by the hands, and the band used merely for the purpose of retention,—as should always be the case,—and supposing, also, that no undue and unnecessary force has been employed, this complaint furnishes no valid excuse for a relaxation of the band. If such indulgence be granted, a retraction of the limb ensues, and in the course of a few hours a renewal of the extension becomes absolutely requisite. For the reasons given in the last paragraph, irritation invariably follows,—the pain is renewed and exacerbated, and the process is usually repeated from time to time. Under such a course of treatment, excoriation and ulceration are rendered almost inevitable, and it is fortunate for the patient if a slough upon the heel is not coupled with a destruction of the cuticle in the perineum by the end of the second week, rendering it impossible to prevent a shortening of the limb from utter intolerance of pressure.

If, upon the first occurrence of such a complaint, a vigorous opiate be administered,—followed, when necessary, by a gentle aperient,—without any intermission of the extension, it will be found that, in a vast majority of cases, the parts will become habituated to the pressure in the course of a few hours, the pain will cease, and all future difficulties of this nature will be removed.

(*To be continued.*)

